# Innovations in Breast Cancer Surgery and Their Adoption and Adaptation in Iraq

**DOI:** 10.7759/cureus.66506

**Published:** 2024-08-09

**Authors:** Aqeel S Mahmood, Osama Jalal Fakhir, Moustafa A Shakir, Rania H Al-Taie, Mustafa Ismail

**Affiliations:** 1 Department of Surgery, College of Medicine, University of Baghdad, Baghdad, IRQ; 2 Department of Surgical Oncology, Oncology Teaching Hospital, Baghdad, IRQ; 3 Department of Surgery, Martyr Dhari Al-Fayad Hospital, Baghdad, IRQ; 4 Department of Surgery, College of Medicine, University of Mustansiriyah, Baghdad, IRQ

**Keywords:** iraq, radiotherapy, breast-conserving surgery, neoadjuvant chemotherapy, breast cancer surgery

## Abstract

Through this editorial, we have attempted to provide an update on the changing scenario for breast cancer surgery in Iraq by describing giant steps toward the adoption of new treatments. One factor to consider is the general trend towards neoadjuvant chemotherapy (NACT) and breast-conserving surgery (BCS) in regions such as Kurdistan, which indicates a preference for these minimally invasive approaches. Additionally, new perspectives on multifocal breast cancer in Baghdad demonstrate that BCS can be effective, with local recurrence rates comparable to mastectomy. Radiotherapy, particularly hypofractionated three-dimensional conformal radiotherapy (3DCRT), has shown substantial benefits in local control and progression-free survival. The importance of timely surgical interventions is also emphasized; most Iraqi women who receive a mastectomy stress to go through surgical interventions within three months of diagnosis. All these are significant reasons for optimism with regard to attaining more exemplary outcomes in patients as well as good strides toward international best practices. Such steps show that Iraq is keen on incorporating advanced surgical techniques that ameliorate breast cancer management.

## Editorial

Background

Breast cancer remains a formidable challenge in Iraq, as it is the most common malignancy among Iraqi women and a leading cause of cancer-related deaths. The high incidence and mortality rates are compounded by issues such as late-stage diagnosis and limited access to advanced medical treatments. But there have been glimmers of hope lately, with new strategies in surgery and treatments surfacing for breast cancer. Innovations in surgical procedures, better radiotherapy options, higher understanding of treatment, and strategies for dealing with patients are paving the way for improved outcomes. These advancements are transforming the landscape of breast cancer care in Iraq, offering new possibilities for early detection, effective treatment, and enhanced survival rates. This editorial explores the adoption and adaptation of these innovations in Iraq, highlighting key studies that shed light on this transformative journey and the ongoing efforts to improve the quality of breast cancer care in the region.

The many problems connected to breast cancer in Iraq interfere with the diagnosis and treatment of the disease, as well as the welfare of the patients. There is usually a cultural stigma attached to this disease, mainly causing delays in diagnosis and treatment because not many women would like to share their condition with their communities out of fear of social ostracism and judgment. There are also several kinds of wrong information and pieces of traditional knowledge treating breast cancer as a shame. Economically, treatment costs are significantly high for most patients due to poor health insurance coverage, rendering most of them incapable of affording diagnostic tests, surgeries, and continued therapy. The healthcare infrastructure in Iraq exacerbates the problems, with disparities in the delivery of quality care between urban and outlying areas, leaving many patients without the needed support and treatment options. Moreover, the psychological burden is just as heavy; probably, most patients with breast cancer do not have quality mental health services and social support systems. Comprehensive strategies toward addressing these wide-ranging challenges need to take the form of public awareness campaigns, better financial aid, and improvements in healthcare services for the benefit of the patients [1,2].

Embracing neoadjuvant therapy and breast-conserving surgery

The Kurdistan region of Iraq has gone through a series of improvements in nonmetastatic breast cancer care, ranging from escalated neoadjuvant chemotherapy (NACT) to breast-conserving surgery (BCS) applications. A significant study by Namiq and Sulaiman reported a significant increase in the use of NACT from 8.3% to 14.2% in the year 2021 and a simultaneous rise in BCS rates from 36.3% to 43.7% over the same period [1]. This shift towards more conservative surgical approaches is in line with international treatment guidelines, which advocate for the reduction of surgical morbidity while maintaining oncological safety. The increasing trend of BCS, particularly in cases of locally advanced breast cancer (LABC), reflects a growing commitment within the region to embrace less invasive yet effective treatment modalities. This transition has been facilitated by the broader use of NACT, which helps to downstage tumors, making them more amenable to breast conservation. The amalgamation of all these practices additionally supports the role of multidisciplinary team case discussions, continuing professional education for providers, and comprehensive patient information programs to work in the same direction to optimally achieve quality care for breast cancer patients and to assure that patients receive personalized, state-of-the-art treatment.

Addressing multifocal breast cancer

Multifocal (MF) breast cancer, characterized by multiple tumors within the same quadrant, has traditionally been considered a contraindication for BCS due to the perceived higher risk of local recurrence. However, a groundbreaking study from Baghdad challenges this conventional view. Mahmood et al. conducted a study involving 239 patients diagnosed with MF breast cancer, finding that those who underwent BCS experienced a local recurrence rate of just 1.37%, which is comparable to the 1.20% recurrence rate observed in patients who underwent mastectomy [2]. This study has opened up new avenues, in which careful selection of patients and thorough surgical techniques would make BCS feasible even in MF breast cancer cases. Results, for the same reasons, argue in favor of revisiting surgical strategies geared towards invasive procedures and, at the same time, avoiding ones that might compromise oncological outcomes. This shift not only supports the psychological and aesthetic benefits associated with BCS but also aligns with a global movement towards more personalized and patient-centered cancer care. By leveraging local data and experiences, Iraqi oncologists are contributing valuable insights into the international discourse on breast cancer treatment.

Enhancing survival with adjuvant radiotherapy

Radiotherapy is one of the most essential components of breast cancer treatment concerning local control and progression-free survival (PFS) improvement. Al-Naqqash et al. assessed the effects of hypofractionated three-dimensional conformal radiotherapy (3DCRT) on Iraqi breast cancer patients. The research involved 299 women treated at the Baghdad Radiation Oncology Center, and the results were compelling. The study found a high frequency of relapse-free survival, with adjuvant radiotherapy significantly reducing locoregional recurrence and distant metastasis [3]. These findings underscore the efficacy of modern radiotherapy techniques in managing breast cancer. The use of 3DCRT, which allows for precise targeting of tumor sites while sparing the surrounding healthy tissue, represents a significant advancement in radiotherapeutic practices. By adopting such innovations, Iraqi oncology centers are not only improving survival rates but also enhancing the quality of life of breast cancer patients. This approach highlights the importance of integrating advanced technologies and evidence-based practices into the national cancer treatment protocols, ensuring that patients receive the best possible care.

Timeliness in surgical intervention

The time from diagnosis to surgical intervention is a very crucial aspect of breast cancer management that directly impacts the patient outcome. In this regard, Alwan et al. evaluated the time lag in Iraqi women who had mastectomy. In this study involving 226 patients, findings showed that 67.7% of patients had undergone mastectomy during the initial month after diagnosis, and a significant proportion of patients (92%) had surgery within three months [4]. These findings highlight the critical importance of reducing delays in surgical intervention. Prompt surgical treatment is associated with better prognosis and reduced risk of cancer progression. The study emphasized the need for robust healthcare infrastructure and streamlined processes to ensure timely access to surgical care. Public awareness campaigns regarding early diagnosis and timely treatment should go hand in glove to ensure high-quality outcomes in breast cancer management in Iraq. It is important to note that solving such issues might enable the healthcare system to improve radically in terms of survival rates and, generally, more effective breast cancer management.

Advances in surgical techniques

Recent surgical techniques for the treatment of breast cancer have improved patient outcomes to a remarkable extent and have significantly decreased surgical morbidity in Iraq. Of most significance is breast-conserving surgery, which is being done more and more frequently. Studies conducted by Namiq and Sulaiman demonstrated unbelievable BCS rates that reflect not only adaptation to more conservative forms of surgical intervention but also the development of less invasive surgical options that are still oncologically sound yet translate into good cosmetic results [1,3]. Higher precision and effectiveness in surgical interventions have further been achieved in the recent past through the incorporation of advanced technologies, such as intraoperative imaging and robot-assisted surgery. These technologies allow not only more exact resections to be performed by the surgeon but also, in effect, more apparent small margins post-resection, and thereby reduce local recurrence. Better aesthetic results have thus come about for many patients due to oncoplastic surgery. The Iraqi healthcare system's commitment to these innovative surgical practices significantly contributes to aligning with international standards and advancing breast cancer care.

Access to care and patient support

Infrastructure improvement within healthcare is critical for extending advanced surgical care options to all locations, including very rural and underserved areas. Specialized breast cancer centers and mobile clinics may also serve to reduce this gap in healthcare delivery and bring about timely and high-quality care to all patients, regardless of their place of abode. Such urgent steps are essential in optimizing treatment outcomes in a lineup of diagnostic and referral processes for breast cancer care in Iraq that are similar to the best global practices [4,5].

To address the psychosocial stress of a cancer diagnosis, support groups or other counseling services must be established to provide emotional and mental health counseling to these patients. These aid the patients to go through much of the tension and worry associated with the treatment process in a better way, uplifting the patients and assuring them that they belong to a community and share similar experiences. On one hand, rehabilitation programs that minimize the physical impacts that occur after surgery and during treatment include various types of physiotherapy to ease a patient's mobility ailments and minimize the degree of lymphedema, which is a notable complication following breast cancer surgery. On the other hand, health promotion and education programs designed to support patients with the help of information related to lifestyle modifications, exercise, and necessary nutrition prescriptions contribute substantially to the recovery process and the quest for positive health results [4,5].

Supportive care must reach into the realm of palliative services, which help with pain management and alleviation of symptoms in patients with end-stage diseases. The integral approach to the care of patients in Iraq emphasizes not only the physical aspects of recovery but also the emotional and social well-being of patients. Altogether, it is through these holistic rehabilitation/social support plans that the healthcare system in Iraq makes a difference for the breast cancer patients in their treatment journey.

Healthcare policy and funding

The landscape for providing breast cancer care in Iraq has also been influenced over the years by health policies and funding initiatives that directly target the enhancement and improvement of accessibility and quality of treatment. To our knowledge, historically, the healthcare system of Iraq was one of the best in the Middle East. Unfortunately, successive wars, sanctions, and political instability greatly impaired the health infrastructure and caused shortages in some medical resources, expertise, and funding. To resolve these difficulties, the Ministry for Health has been initiating reformatory and collaborative activities. This includes the establishment of the Iraqi Cancer Board in 1985 and the formation of a National Cancer Control Plan in 2010, which focused on making the processes of cancer diagnoses, treatment, and palliative care better and more efficient.

In cancer care, international collaborations with many organizations, including the World Health Organization (WHO), International Atomic Energy Agency (IAEA), and with regional institutions, have facilitated the overall experience and technology transfer. These collaborations include meetings and training programs for the health system personnel in areas like medical physics, radiation safety, and oncology and visits to institutions of advanced treatment for cancer. Despite these efforts and investments, the healthcare system is grappling with the cited challenges. Campaigns have been carried out to create awareness among the public to reduce stigmatization, promote early screening, and encourage on-time treatment to improve stigmas. It could be concluded that considerable progress has been noticed in the field of health policy and financing for improvement in breast cancer care in Iraq. Still, consistent efforts are to be made from time to time to provide better quality care. Continued investment in health infrastructure, expanding health insurance coverage, and continuing international collaborations will be essential for empowerment against the prevailing challenges and a better landscape for breast cancer care in Iraq [5].

Conclusion

The Iraqi experience with breast cancer surgery and treatment showcases significant strides towards adopting innovative practices. The increasing use of neoadjuvant therapy, breast-conserving surgery, and advanced radiotherapy techniques, coupled with a focus on timely intervention and comprehensive care, reflects a commitment to improving patient outcomes. As these innovations continue to take root, they offer a beacon of hope for breast cancer patients in Iraq, underscoring the importance of continuous adaptation and improvement in cancer care.

## References

[1] Namiq KS, Sulaiman LR (2023). Neoadjuvant therapy in nonmetastatic breast cancer in Kurdistan, Iraq. JCO Glob Oncol.

[2] Mahmood AS, Altameemi E, Shakir AA, Sabri S, Ismail M (2023). The challenges of breast conservative surgery in multifocal breast cancer: the first insights from Iraq experience. Indian J Med Sci.

[3] Al-Naqqash M, Al-Bdaer EK, Saleh WA, Al-Shewered AS (2019). Progression free survival in Iraqi breast cancer patients treated with adjuvant 3D conformal radiotherapy: a cross-sectional study. F1000Research.

[4] Alwan N, Tawfeeq FN, Sattar SA, Yihya F (2019). Assessing the period between diagnosis of breast cancer and surgical treatment among mastectomized female patients in Iraq. Int J Med Res Health Sci.

[5] Al Alwan NA (2022). General oncology care in Iraq. Cancer in the Arab World.

